# Fabric Tactile Prediction Method Based on Spider Diagram

**DOI:** 10.3390/s25103187

**Published:** 2025-05-19

**Authors:** Ruifeng Xie, Shuyang Ding, Zeyu Cheng, Luowei Ma, Yanzhu Yang

**Affiliations:** College of Mechanical Engineering, Donghua University, Shanghai 201620, China; 2231205@mail.dhu.edu.cn (R.X.); shuyangding@dhu.edu.cn (S.D.); 15053387302@163.com (Z.C.); 2231162@mail.dhu.edu.cn (L.M.)

**Keywords:** fabric touch, textiles, fabric physical properties, predictive modeling

## Abstract

The detection and quantification of fabric tactile sensations are crucial in textile production and marketing as they are closely linked to textile comfort and serve as key criteria for consumers when selecting fabrics. Previous studies have predominantly focused on measuring the physical properties of fabrics, often neglecting correlations between these parameters and tactile sensations. This oversight complicates customers’ ability to assess the tactile experience of fabrics during online purchasing. This study first obtained subjective evaluations of three types of fabric tactile sensations through experiments involving volunteer participants. Subsequently, five objective physical properties that characterize fabric tactile properties were proposed and experimentally tested on 15 fabric samples categorized by yarn weight, weave pattern, and material. A fabric tactile spider diagram was created by normalizing the values of the five physical properties across the 15 fabric samples. The grading of the physical properties was then performed based on the proposed evaluation index. These spider diagrams were compared with the subjective evaluation results to analyze the physical properties that most significantly influenced subjective perception, ultimately leading to the development of a highly reliable fabric touch prediction model.

## 1. Introduction

Textiles are essential in daily life. People often judge clothes based on visual and tactile impressions [1,2], typically by looking, touching, and trying them on. However, in the era of big data and cloud computing, lifestyles have undergone significant changes. With improving economic standards, clothing needs have shifted from basic coverage and warmth to a focus on health and comfort. Shopping has also shifted to online platforms. When selecting clothes online, people rely on photos and descriptions, which are often unreliable [3]. Thus, there is an urgent need for a fabric feel evaluation system to enhance the online clothing shopping experience.

With the development of textile technology, the quantification and evaluation of fabric feel have attracted considerable attention, and many scholars have conducted relevant research [4,5,6]. In 2014, SDL Atlas and the Hong Kong Polytechnic University jointly developed the Fabric Touch Tester (FTT) [7,8,9], which measures various properties of fabrics to generate 18 indices. These indices are then utilized in a quality assessment model to output the softness, smoothness, and warmth of the fabric. Esra et al. [10] tested denim fabrics of different fiber types, weaves, and weft densities using the FTT and sensory evaluation questionnaires and analyzed the relationship between their physical properties and tactile comfort.

In addition to the FTT, other machine-tested physical properties of fabrics also support the quantification of fabric feel [11,12]. Kura et al. [13] used the Kawabata (KES-FB4) testing instrument to measure the surface roughness of fabrics and developed a linear regression model to predict it, which showed a strong correlation with the actual measured values within a 95% confidence interval. Yawen et al. [14] objectively evaluated the tactile comfort of knitted fabrics using the curve and structure parameters of the ring-shaped style tester, confirming the effectiveness of the tester’s parameters.

In prior research, a human-like fabric detection method based on tactile sensors [15] was proposed. Applied to 21 sample groups, the method explored fabric material properties by analyzing the energy spectrum integral *S*(*FFT*^2^) and the spectrum integral *S*(*FFT*) of vibration signals. The results showed that the energy spectrum integral slope *Kv* and the spectrum integral slope *Kw* are effective for characterizing fabric surface roughness and hardness.

In this study, we introduced bendability *B*, elasticity *K*, and thermal conductivity ∆*K* as physical properties to characterize the bending, stretching, and thermal characteristics of fabrics, respectively. This expanded the original two-dimensional data into five dimensions. We tested 15 fabric samples based on these properties. Using the test results, we constructed a spider diagram of the fabric physical properties and translated abstract physical property values into intuitive and comprehensible property levels, thereby providing an efficient tool for quantifying fabric physical properties.

Additionally, volunteers participated in a subjective evaluation experiment assessing the tactile feel of the 15 fabric samples. We fitted the spider diagram to the subjective evaluation results to explore the impact of individual physical properties on subjective evaluations. This approach successfully built a model for predicting fabric feel based on the physical property spider diagram, offering a simple and reliable method for estimating fabric tactile feel.

## 2. Experimental and Method

### 2.1. Fabrics Samples

Given the extensive diversity of fabric styles, achieving comprehensive coverage in fabric sample selection remains unfeasible. Therefore, this study selected fabric samples from three distinct perspectives to encompass commonly encountered fabrics and to derive more reliable conclusions. The first perspective considered fabrics composed of the same material but exhibiting varying yarn weights. Fabrics with lighter yarn weights typically exhibit a softer, more delicate feel and are suitable for summer garments or applications requiring enhanced breathability. In contrast, fabrics with heavier yarn weights offer a thicker, warmer feel, rendering them appropriate for winter garments or applications demanding greater insulation. This variation in yarn weight was incorporated to evaluate alterations in tactile perception. The second perspective examined fabrics composed of the same material but constructed with different weave patterns, such as plain and twill. These weave patterns vary in terms of sparsity and density, thereby producing distinct tactile textures. The third perspective considered fabrics comprising different materials, including both single-component and composite fabrics, to investigate how material composition influences tactile perception.

**Figure 1:**
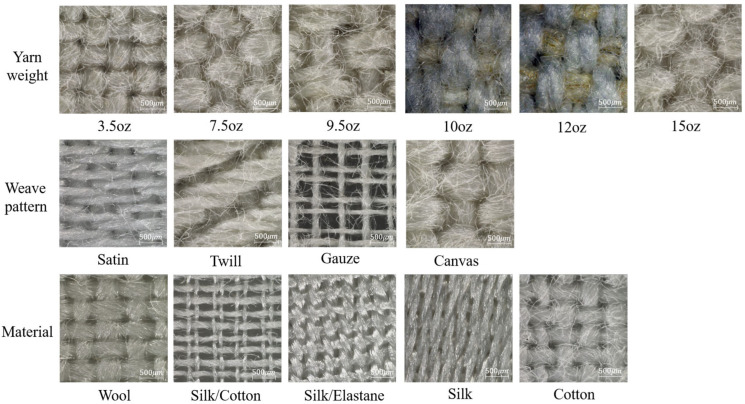
The 15 fabric samples taken by super-depth-of-field microscope.

Figure 1 shows a texture image of a fabric sample taken by super-depth-of-field microscope. A total of 15 different commercial textiles produced by Whaleys (Bradford) Ltd. (Bradford, UK) were used in this study. These included 6 cotton canvas fabrics with yarn weights ranging from 3.5 to 15 oz/yd^2^; 4 cotton fabrics with different weave patterns of canvas, gauze, satin, and twill; and 5 plain fabrics made of different materials including 3 natural materials, cotton, silk and wool, and 2 composites: silk and elastane; and silk and cotton. All these fabrics were precisely cut into 8 cm × 8 cm rectangular samples.

Following the selection of the fabric samples, systematic experiments will be conducted to analyze their physical properties. Initially, a subjective tactile evaluation will be performed, where volunteers will rate the cool-warm feeling, smooth feeling, and soft feeling of the fabrics. Subsequently, an experimental apparatus will be employed to measure five physical properties: hardness, roughness, bending, tensile, and thermal properties. These results will serve as the foundation for analyzing the effects of yarn weight, weave pattern, and material variations on fabric tactile properties, and will provide essential data for subsequent touch prediction modeling.

### 2.2. Instruments

#### 2.2.1. Subjective Testing Platform

All haptic experiments were conducted under controlled environmental conditions, specifically at a room temperature of 22 °C and a relative humidity of 35–40%. To minimize external interference, an 80 cm × 80 cm × 80 cm LED light photographic tent was set up at the experimental site, accompanied by a set of tables and chairs. A white plastic backing board was placed directly in front of the participant within the tent to eliminate visual distractions. Additionally, participants were instructed to wear noise-canceling headphones to mitigate the influence of ambient noise on their judgment (Figure S1).

#### 2.2.2. POD (Pin on Disk) Tribometer

**Figure 2:**
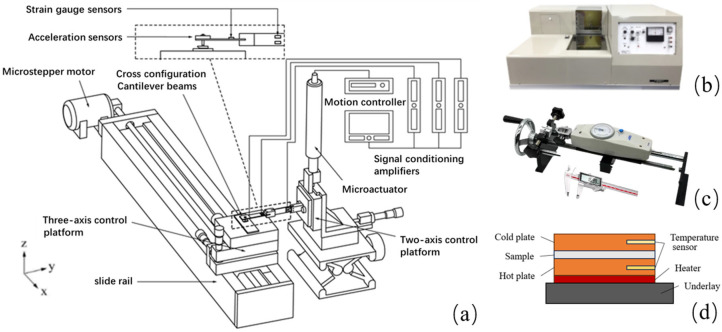
(**a**) POD tribometer. (**b**) KES-FB2-A Pure Bending Tester. (**c**) Tensile property testing device. (**d**) Thermal Characterization Testing Device.

As shown in Figure 2a, the device comprises an electrically controlled movable platform (Beijing PDV Instrument Co., Ltd., Beijing, China) and a set of sensor modules. The fabric sample to be tested is attached to the upper surface of a three-axis adjustable platform. Positioned above is a cross-shaped cantilever beam, fitted with a detachable pin-holder that holds a steel ball pin (diameter = 6 mm, Ra = 0.05 μm). Controlled by a micro-driver, the cantilever beam moves vertically along the *z*-axis, bringing the pin into contact with the fabric sample. Upon contact, the three-axis adjustable platform, driven by a brushless stepper motor (Y07-43D1-4275, Shinano Motor Co., Ltd., Nagano, Japan), moves along the x-axis. This movement causes friction between the fabric and the pin, simulating the human touch process. Positioned on the cantilever beam above the pin is a three-axis capacitive accelerometer (ADXL335, ADI, Shanghai, China), which collects vibration signals during friction. It has dimensions of 4 mm × 4 mm × 1.45 mm, weighs 1.27 g, features an acceleration measurement range of ±3 g, and offers frequency bandwidths of 0.5 Hz to 1.6 kHz (x and y axes) and 0.5 Hz to 550 Hz (*z*-axis), with a three-axis sensitivity of 300 ± 0.001 mV/g. At the fixed ends of the two cantilever beams, a Wheatstone full-bridge strain gauge sensor (with a strain factor of 115) is connected in parallel. This sensor measures both the vertical load and horizontal frictional force. After collection, the data passes through three differential amplifiers (AD-630, Shanghai, China) and is subsequently routed to the NI-SCC-68 DAQ data acquisition card (NI, Austin, TX, USA). The data are transmitted to LabView via temperature–drift–resistant sensors and NI anti-electromagnetic-interference wires, and is ultimately displayed on a self-designed three-channel signal acquisition program interface.

#### 2.2.3. KES-FB Fabric Style Analyzer

The KES-FB system [16], developed by KATO TECH Co., Ltd., is an apparatus designed to assess fabric feel and style characteristics. This system evaluates fabric style by analyzing mechanical properties that correspond to style characteristics. Quantitative indices are derived using mathematical methods, with a primary focus on the fabric’s deformation under minimal loads and low stress. In this study, the KES-FB2-A pure bending tester (KATO TECH Co., Ltd., Kyoto, Japan) was utilized to measure the bending properties of fabrics (Figure 2b).

#### 2.2.4. Self-Made Testing Device for Fabric Tensile Properties

As shown in Figure 2c, the device comprises a horizontal tensile test fixture with pointer force meter (Dongguan Fuma Electronic Equipment Co., Ltd., Dongguan, China), and a vernier caliper (Qingdao Tesco Hardware Tools Co., Ltd., Qingdao, China). The horizontal tensile fixture is used to secure the fabric sample and the pointer force meter. The pointer force meter measures the tensile force applied to the fabric during stretching, while the vernier caliper measures the deformation of the fabric sample.

#### 2.2.5. Self-Made Testing Device for Thermal Properties of Fabrics

As shown in Figure 2d, the device comprises a heating plate (Jiangyin Jinyu Electric Heating Electric Appliance Co., Ltd., Jiangyin, China), two thin copper sheets, and a temperature sensor. The lower copper sheet, in contact with the heating plate, serves as the hot plate, while the upper copper sheet acts as the cold plate. During testing, a fabric sample is placed between the hot and cold copper sheets. Two thermometers are positioned on the copper plates, close to the fabric surface, in order to measure the temperatures.

### 2.3. Experimental Procedure and Method

#### 2.3.1. Fabric Touch Evaluation Test

The subjective evaluation of fabrics is typically conducted by experienced inspectors, who assess the tactile properties of the fabric based on personal judgment [5,17]. Inspectors generally follow a four-step process—pinch, touch, grasp, and observe—to make an initial assessment of the fabric’s characteristics. The terminology used in subjective evaluation includes descriptors such as hard/soft, rough/smooth, fine/coarse, sticky/slippery, cold/warm, and so forth. Although this method is straightforward and efficient, it is susceptible to biases introduced by factors such as the inspector’s social background, environment, gender, age, and personal preferences. To mitigate these biases, it is recommended that the number of evaluators be increased. In this study, 50 volunteers were randomly selected to subjectively evaluate the fabric based on the cool-warm feeling, smooth feeling, and soft feeling.

Prior to the commencement of the experiment, volunteers washed their hands using glycerin soap and dried them with paper towels to mitigate the potential impact of sweat on tactile perception. They then wore noise-canceling headphones and seated themselves quietly inside the tent. The experiment prepared five sets of identical fabric samples, with one set used for testing by every ten volunteers. Volunteers performed a series of actions on each group of fabric samples, which includes (1) sliding the surface of the fabric back and forth with the index finger of their dominant hand; (2) holding a corner of the fabric with the index finger and thumb at both upper and lower positions, pressing the index finger against the thumb to assess tactile sensation; (3) grasping the fabric with both hands and tugging it slightly several times; and (4) touching the fabric freely without restriction. This process continued until all fabric samples were evaluated. Finally, the average scores from the 50 volunteers were calculated for each fabric based on subjective evaluations of the cool-warm feeling, smooth feeling, and soft feeling. These averages were then used to derive a comprehensive subjective evaluation score for the fabric samples.

To further validate the subjective evaluations and quantitatively analyze the tactile properties of fabrics, we employed a standardized experimental apparatus to systematically measure five physical properties: hardness, surface roughness, bending properties, tensile properties, and thermal properties. These measurements provide an objective quantification of the fabrics’ physical properties.

#### 2.3.2. POD Tribometer Experiment

In this experiment, each sample group was tested using a POD tribometer. The motorized platform was precisely programmed to simulate manual fabric friction under two operational conditions: (i) a constant positive pressure of 0.15 V with sliding speeds ranging from 5 mm/s to 40 mm/s; and (ii) a constant sliding speed of 20 mm/s with positive pressures varying between 0.05 V and 0.25 V. During motor operation, the friction instrument’s probe contacts the test fabric, generating vibrational signals acquired via a LabVIEW-based system. Energy spectral analysis and integration were applied to the constant normal pressure signal, while frequency spectrum analysis and integration were performed on the constant sliding velocity signal (both in MATLAB 2020b), yielding the following expression [18]:

(1) $$S(FFT)=\frac{\left(b-a\right)}{2N}\sum_{i=1}^{N}\left[FFT(f_{i})+FFT(f_{i+1})\right]$$

(2) $$S(FFT^{2})=\frac{\left(b-a\right)}{2N}\sum_{j=1}^{N}\left[FFT{\left(f_{i}\right)}^{2}+FFT{\left(f_{i+1}\right)}^{2}\right]$$

Here, *FFT*(*f_i_*) denotes the fast Fourier transform of the time-domain variable *x*(*t*). The indices *i* and *j* range from 0 to *N*, where *N* represents the number of sampling points, and the spacing between each point is (b−a)/N. *S*(*FFT*) denotes the integral of *FFT*(*f_i_*), while *S*(*FFT*^2^) denotes the integral of *FFT*(*f_i_*)^2^.

The vibration signals were divided into fixed-length segments. Each segment was processed in MATLAB to calculate *S*(*FFT*) and *S*(*FFT*^2^). These values were averaged across all segments, obtaining *S*(*FFT*) and *S*(*FFT*^2^) with minimized experimental error under the given conditions. The independent variable is defined as positive pressure, and *Kw* is the slope of the least-squares fitted line for *S*(*FFT*) as a function of this variable. Similarly, when the independent variable is sliding velocity, *Kv* is the slope of the least-squares fitted line for *S*(*FFT*^2^) as a function of this variable.

#### 2.3.3. Bending Experiment

At the beginning of the experiment, the sample is clamped between the two collets of the KES-FB2-A pure bending tester. The test is then initiated by operating the control panel in accordance with the instrument’s instructions. During the test, one of the collets swings back and forth in an arc with a curvature range of −90 to 90 degrees. The strain curvature of the sample and the bending moment are measured using a potentiometer and a strain torque meter. Subsequently, a coordinate curve is plotted with the bending moment on the *x*-axis and the bending rate on the *y*-axis. Each curve is linearly fitted using the least squares method and the slope of the fitted line is recorded as *B*.

#### 2.3.4. Tensile Experiment

In this experiment, the fabric sample is secured at both ends using a fixture. The turntable is then slowly rotated while monitoring the dynamometer’s pointer. The rotation continues until the pointer approaches the zero position, indicating that the fabric sample has been flattened. Vernier calipers are used to measure and record the length $L_{0}$ of the clamped portion of the fabric, which represents the initial length before stretching. The turntable is then rotated until the pointer indicates *F* = 20 N. At this point, the length L of the clamped portion of the fabric is measured again using the vernier calipers, representing the length after stretching. The coefficient of elasticity for each sample is calculated using the following formula:

(3) $$K=\frac{F}{L-L_{0}}$$

#### 2.3.5. Heat Conduction Experiment

In a room maintained at 22 °C, fabric samples are placed between the upper and lower copper plates of a homemade thermal properties detection device. The initial temperature of the copper plates $t_{0}$ is recorded. The heating plate is then activated, and after a heating period of *T* = 2 min, the temperatures of the hot plate and the cold plate are measured and recorded as $t_{1}$ and $t_{2}$, respectively. After turning off the heating plate, the device is allowed to cool back to the initial temperature $t_{0}$ before measuring the next sample.

The average warming rates of the hot and cold plates during the test were

(4) $$K_{1}=\frac{t_{1}-t_{0}}{T}$$

(5) $$K_{2}=\frac{t_{2}-t_{0}}{T}$$

Differential rate of warming:

(6) $$\Delta K=K_{1}-K_{2}=\frac{t_{1}-t_{2}}{T}$$

## 3. Results and Discussions

### 3.1. Fabric Touch Evaluation Experiment

**Figure 3:**
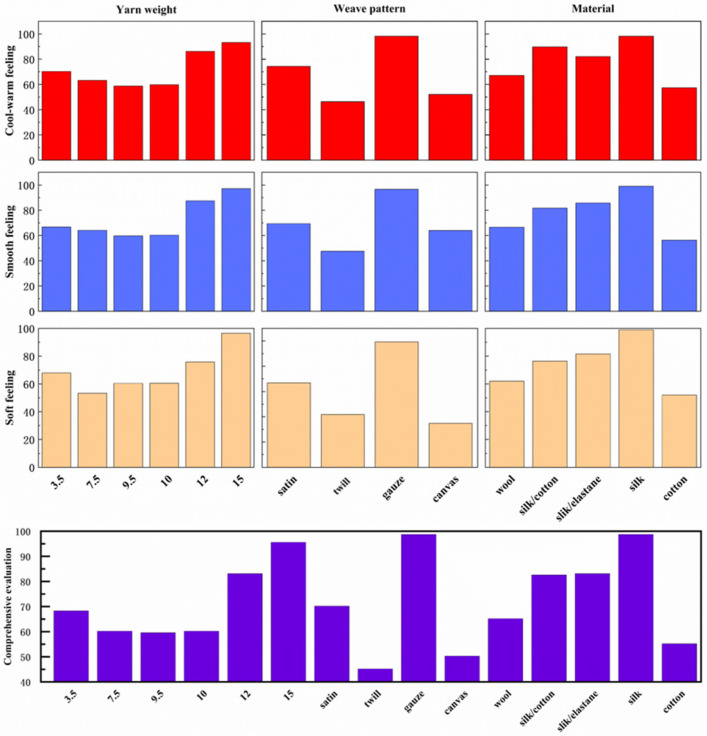
Touch evaluation experiment results. The upper nine-square grid is the evaluation value of the three types of fabrics: cold-warm, smooth, and soft feeling. The image in the lower column is the comprehensive evaluation value.

As shown in Figure 3, there are notable differences in the subjective evaluations of fabrics with varying yarn weights. The 9.5 oz/yd^2^ fabric samples received the lowest overall subjective ratings, while the 15 oz/yd^2^ samples received the highest. Similarly, subjective evaluations varied with different weave patterns: twill fabrics were rated the lowest, and gauze fabrics were rated the highest. Differences were also observed in subjective evaluations based on fabric materials: cotton had the lowest overall rating, whereas silk had the highest. These results suggest that fabrics with higher overall ratings in the selected batch also tend to excel in their specific tactile attributes. Thus, using a composite evaluation to represent subjective evaluations of fabrics is both intuitive and reliable.

### 3.2. Construction and Analysis of Physical Property Spider Diagrams

Through four measurement experiments, the values of five physical properties for evaluating the texture of 15 fabric samples were obtained, as shown in Table 1. From the table, it is evident that some physical properties of the fabrics are prominent, such as *Kv* and *Kw*, which increase simultaneously with the increase in yarn weight.

**Figure 4:**
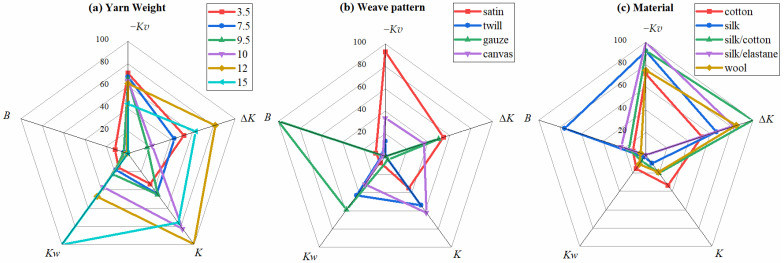
Spider diagram drawn from experimental data. (**a**) Six different yarn weights. (**b**) Four different weave patterns. (**c**) Five different materials.

The physical property values in Table 1 were subjected to normalization, and spider diagrams were plotted accordingly. In the normalized data, −*Kv* indicates the opposite magnitude of the actual *Kv* value. *Kv* and *Kw* are linked to the roughness and hardness of fabrics, respectively. In this study, these values are used to represent fabric roughness and hardness. On the spider diagram, a larger −*Kv* value indicates a smoother fabric surface, while a larger *Kw* value signifies that the fabric is more compressible, i.e., fuller. The bending rigidity of the fabric is represented by *B*, which is experimentally obtained from the KES-FB2-A Pure Bending Tester. On the spider diagram, a larger *B* value implies greater bendability of the fabric, meaning it will exhibit a higher bending rate under a given bending moment. In the tensile experiment, the coefficient *K* is obtained by measuring the elastic modulus of the sample, reflecting the fabric’s tensile elasticity. On the spider diagram, a larger *K* value indicates greater elastic deformation under constant stress, signifying better elasticity of the fabric. Each of the different forms of heat transfer influence the heat transfer process of fabrics in distinct ways. Based on the heat transfer mechanism described by Dun et al. [19], this experiment uses the rate of temperature rise over a specific period to assess the fabric’s overall thermal conductivity. As depicted in the spider diagram, a larger value of Δ*K* indicates a higher temperature rise in the cold plate heated by the fabric, reflecting better thermal conductivity. The spider diagram presents five physical properties in the following order: −*Kv*, Δ*K*, *K*, *Kw*, and *B*, as shown in Figure 4. Although this ordering method is not the only one possible, it provides the most reliable fit between physical properties and subjective evaluation results, as discussed in Section 3.4.

Figure 4 shows that the 15 fabric samples each display distinct spider diagram shapes, with the five physical properties influencing the tactile sensation of each fabric sample clearly shown in the spider diagrams. In Figure 4a, the 12 oz and 15 oz fabrics show larger areas, indicating that these two fabrics have larger multiple physical properties compared to the other fabrics in the same group. When comparing these two fabrics, it can be seen that the *Kw* of the 15 oz fabric is significantly larger than that of the 12 oz fabric, giving it a more easily compressible physical property. As the weight of the yarn made of the same material increases, the spider diagrams of the fabrics in Figure 4a are similar in shape but differ in area size. As shown in Figure 4b, when the materials are the same, different weave patterns can lead to significant differences in the measured physical properties, thereby changing the shape of the pattern. This indicates that the weave pattern is an important factor affecting the physical properties of fabrics. Figure 4c shows that changing the material also affects the physical properties of the fabric. However, unlike changing the weave pattern, it does not significantly alter the shape of the pattern but mainly leads to changes in the area of the pattern. By comparing the three groups of spider diagrams, we can intuitively understand the differences in the physical properties of different fabrics and can quickly distinguish fabrics with different textures through their touch spider diagrams.

This study proposes a set of grading criteria designed to streamline normalized fabric property values into clear and intuitive grading indicators for assessing physical properties. Given that minor numerical variations within the 0–100 normalized range do not consistently correlate with perceptible physical differences, combined with inherent measurement uncertainties, we have implemented a decile-based grading system. Under this framework, fabrics within the same grade exhibit comparable physical characteristics, while distinct grades demonstrate discernible differences. The grading criteria are detailed in Table 2. Next, the physical property concepts in Table 2 will be introduced.

Roughness: the surface roughness of a fabric, characterized by the slope *Kv* of a fitted straight line of a function, which consists of the integral of the energy spectrum of the vibrational acceleration of the sample under constant positive pressure with respect to the sliding velocity. A higher roughness level indicates a rougher surface, and vice versa for a smoother surface.

Fullness: Fullness refers to a fabric’s ability to deform under compression, which is quantified by the slope of a fitted straight line representing the function *Kw*. This function is derived from the spectral integral of the vibrational acceleration of the sample at a constant sliding speed under positive pressure. A higher degree of compressive deformation at a given pressure results in a higher fullness rating, indicating a fuller fabric.

Bendability: The performance of a fabric subjected to bending deformation, characterized by the slope *B* of the bending rate with respect to the load. The greater the bendability at a constant load, the higher the bendability rating, indicating a softer fabric.

Elasticity: Elasticity refers to a fabric’s response to tensile deformation, quantified by the tensile slope *K* under a fixed tensile load. A steeper slope indicates a higher degree of elasticity, meaning the fabric exhibits greater elasticity.

Thermal conductivity: The performance of the fabric to conduct heat faster or slower, measured as the temperature difference Δ*K* between the hot and cold copper plates after a certain time.

**Figure 5:**
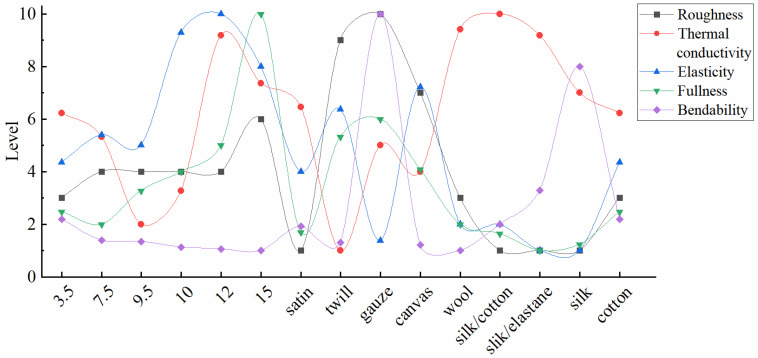
Fabric physical properties grading.

The grading results are presented in Figure 5. After grading simplification, the five physical property grades are clearly depicted in this graph. Unlike the spider diagram, which visually represents individual data points, the physical property grading simplifies the objective data into easily interpretable characteristic grades. For textile mills, the fabrics they produce can be graded for physical properties via the program in this study, thereby more succinctly presenting the physical properties of fabrics when promoting them. Providing physical property grading for fabrics during online shopping offers customers more concrete and objective information about the clothing, as opposed to the subjective and generalized descriptions like “more comfortable”, “softer”, or “smoother” often used by merchants. Additionally, customers can compare the fabric they are currently wearing with the rating of a fabric they intend to purchase online, gaining specific tactile insights from the differences and similarities in the ratings, which aids in making informed clothing choices.

Although the five physical properties provide comprehensive and detailed information, accurately selecting fabrics with optimal tactile sensations remains challenging. When comparing different fabrics based on these properties, each may have its own advantages and disadvantages. Human judgment alone, due to its subjective nature, can lead to inconsistent results, making it difficult to reach accurate and appropriate conclusions [6]. Therefore, it is essential to develop an analytical method that predicts the overall tactile sensation of fabrics based on the five physical properties, while considering the influence of each attribute on the fabric’s tactile sensation.

### 3.3. Degree of Influence of Physical Properties on Subjective Evaluations

Many researchers have previously studied the effect of fabric physical properties on touch [20,21,22], and many results show that these properties affect fabric hand. However, to clarify the degree of impact on comprehensive hand, subjective evaluation results must be combined. Thus, this study proposes a method to analyze the impact of single attributes on subjective comprehensive evaluation based on the spider diagram area. Next, a mathematical model is established.

We developed a mathematical model:

(7) $$y=\frac{1}{2}(a_{1}x_{1}a_{2}x_{2}+a_{2}x_{2}a_{3}x_{3})\sin(\frac{360{}^{\circ}}{5})+b$$

In this model, y represents the result of the subjective comprehensive evaluation, while $x_{1},{\;x}_{2},$ and ${\;x}_{3}$ denote the values of physical properties of the fabrics in sequential order. The parameters $a_{1},a_{2},a_{3}$, and b are to be determined. Sin72° calculates the area between adjacent axes in a pentagon. The term $a_{1}x_{1}a_{2}x_{2}$ represents the weighted product of neighboring attributes ${(a}_{1}x_{1})\;\times \;(a_{2}x_{2})$ with $a_{2}x_{2}a_{3}x_{3}$ following the same rule. Multiplying these by sin72° quantifies their combined influence on evaluations. The repeated $x_{2}$ indicates its dominant role. Equation (7) can be understood as the treatment of the areas of two triangles between three adjacent physical properties on the spider chart in Figure 4.

After selecting the three physical properties, the data were imported into the model for least squares nonlinear fitting, and the resulting R-squared value was evaluated. A higher R-squared value indicates a stronger correlation between changes in the graph area and changes in the composite evaluation, suggesting that the physical property represented by $x_{2}$ has a greater impact on the subjective evaluation.

According to the fitting results presented in Table 3, the R-squared values for *B*, −*Kv*, and *Kw* are higher, indicating that bendability, surface roughness, and fullness are the primary factors influencing the integrated fabric touch.

Furthermore, analyzing the change in R-squared values in the area-fitting comprehensive evaluation model—after excluding one physical property at a time using the spider diagram (Table S1)—revealed the greatest change in R-squared when excluding *B*, *−Kv*, and *Kw*. This finding suggests that these three physical properties have the most significant impact on the overall tactile sensation.

### 3.4. Spider Diagram-Based Prediction for Comprehensive Evaluation

Examination of the area of the corresponding spider diagram for each fabric reveals that a larger spider diagram area indicates that the fabric may have physical properties that are smoother, fuller, more bendable, more elastic, and more thermally conductive. When combined with the results of tactile evaluation experiments, fabrics with larger spider diagram areas received higher comprehensive evaluations compared to those with smaller areas. This correlation implies that the area of the spider diagram is related to subjective tactile evaluations.

Based on the area calculation of the spider diagram, the following prediction model is proposed:

(8) $$y=\frac{1}{2}(a_{1}x_{1}a_{2}x_{2}+a_{2}x_{2}a_{3}x_{3}+a_{3}x_{3}a_{4}x_{4}+a_{4}x_{4}a_{5}x_{5}+a_{5}x_{5}a_{1}x_{1})\sin(\frac{360{}^{\circ}}{5})+b$$

In the spider diagram, $x_{1},x_{2}\ldots x_{5}$ represent the five axes, each corresponding to a physical property. The parameters $a_{1},a_{2}\ldots a_{5}$ denote the influencing weights of these physical properties, b is the compensation coefficient, and y represents the comprehensive evaluation derived from the prediction. As established in Section 3.3, different physical properties exert varying degrees of influence on subjective evaluation. Therefore, assigning distinct weights to each physical property is crucial for achieving more reliable prediction results. Moreover, physical properties do not affect fabric tactile sensation in complete isolation; thus, the interaction between two physical properties is considered by incorporating their product into the model. This approach accounts for the interactive effects of the physical properties on subjective evaluation.

The tactile comprehensive evaluation data and physical property data from the spider diagram were subjected to least-squares nonlinear fitting. Prior to fitting, it is essential to identify the physical properties corresponding to the five axes of the spider diagram in sequence and to determine which interaction effects between physical properties will be used to predict the comprehensive evaluation of fabrics. The reliability of the model was assessed using the R-squared, root mean square error, and average relative error after fitting.

After continuously transforming the ordering of the physical properties on the spider diagram, the final prediction model is obtained as follows:

(9) $$\begin{array}{l} CE=\frac{1}{2}[1.4487(-Kv)\times 0.0222\Delta K+0.0222\Delta K\times 0.0144K+0.0144K\times 2.9987Kw\\ +2.9987Kw\times 0.0265B+0.0265B\times 1.4487(-Kv)]\times \sin72+38.2143 \end{array}$$

where *CE* refers to the comprehensive evaluation projections. The order of the five physical properties in the spider diagram (Figure 4) is −*Kv*, ∆*K*, *K*, *Kw*, and *B*, with corresponding weights of 1.4487, 0.0222, 0.0144, 2.9987, and 0.0265, respectively. The optimal fit was achieved by adding a constant of 38.2143 after calculating the area using these weights. The model exhibits an R-squared value of 0.92761, an adjusted R-squared of 0.87332, a mean square error (MSE) of 20.7871, a root mean square error (RMSE) of 4.5593, and a mean absolute percentage error (MAPE) of 6.121%. This indicates that −*Kv*, ∆*K*, *B*, *Kw*, and *K* explain 87.332% of the variation in subjective ratings, and the errors are within acceptable limits. The spider diagram also reveals that the interaction effects between −*Kv* and ∆*K*, ∆*K* and *K*, *K* and *Kw*, *Kw* and *B*, and *B* and −*Kv* have the most significant impact on the comprehensive evaluation of tactile properties.

**Figure 6:**
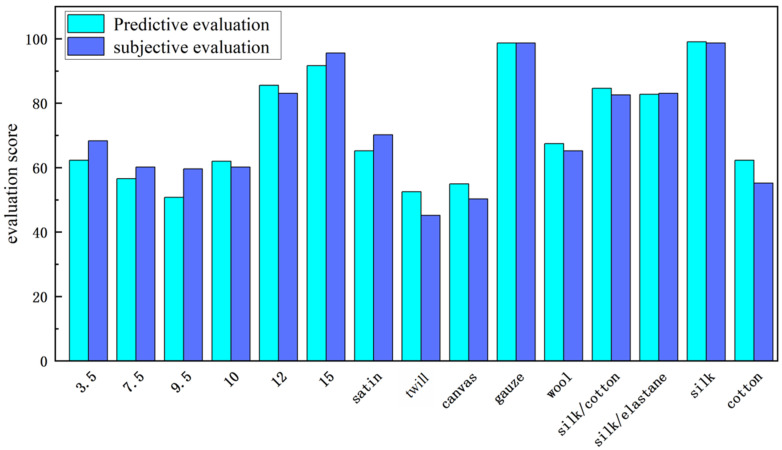
Comparison of physical property prediction results with subjective ratings.

Figure 6 shows that the scores fitted to the physical data of the 15 fabric samples closely match the trends observed in the subjective evaluation scores, indicating a high degree of fit and validating the reliability of the prediction model. Thus, the prediction model based on the fabric tactile spider diagram offers an intuitive, concise, and effective method for fabric tactile detection and evaluation.

## 4. Conclusions

In this study, 15 fabric samples were selected and volunteers evaluated their tactile properties to obtain subjective data. The measured physical properties included hardness, surface roughness, bending properties, tensile properties, and thermal properties. These measurements were obtained using a POD tribometer, KES-FB2-A pure bending tester, custom fabric tensile testing device, and thermal property testing apparatus. The normalized values of these physical properties were used to create spider diagrams, which facilitated the visualization and comparison of tactile characteristics across fabric samples.

The analysis of the spider diagram for individual physical properties revealed a significant positive relationship between fullness (*Kw*), bendability (*B*), and subjective evaluation, a significant negative effect for roughness (*Kv*), and a negligible effect of elasticity (*K*) and thermal conductivity (Δ*K*) on subjective evaluation. Further regression analysis produced a predictive model for subjective evaluation based on the spider diagrams. The model achieves high-precision prediction (adjusted R^2^ = 0.87) by analyzing the interactions between fabric physical properties, particularly the arrangement effects of adjacent properties. Unlike traditional regression models, it maintains high reliability and interpretability while innovatively integrating data analysis with graphical presentation. It can not only capture key influential factors through optimized property arrangement (for example, the tactile score is more reliable when *Kw* is adjacent to *B*), but also reveal complex relationships between properties through intuitive graphics (such as the negative impact of *Kv*).

We propose a spider diagram-based model for predicting fabric tactile sensation, providing a novel tool for textile quantification and evaluation across production, distribution, and consumer shopping. This model facilitates more effective communication of specific tactile information in online shopping environments, thereby enhancing purchase accuracy and consumer satisfaction. Furthermore, it establishes a scientific foundation for textile design and development, enabling manufacturers to optimize product design and production based on consumer preferences and market demands.

## Figures and Tables

**Table 1:** Values for the physical properties of the fabric.

Samples	S(FFT^2^) Slope *Kv*	S(FFT) Slope *Kw*	Bending Property *B*	Coefficient of Elasticity *K*	Differential Rate of Warming Δ*K*
3.5	215.53	3.59	17.20	7272.73	7.45
7.5	242.92	4.15	6.03	9049.77	7.25
9.5	253.48	5.13	5.53	9259.26	6.7
10	267.85	7.86	2.57	15,625	6.8
12	282.8	9.81	1.51	18,518.5	8.1
15	412.56	20.14	0.61	14,492.8	7.7
canvas	486.41	6.72	3.79	12,121.2	7.1
twill	632.55	9.11	5.01	10,695.2	6.3
gauze	727.61	12.12	139.61	2259.89	7.4
satin	64.39	2.08	13.41	7547.17	7.5
silk	69.97	1.16	105.94	3149.61	7.75
silk/cotton	71.43	1.96	22.42	4975.12	8.5
silk/elastane	15.41	0.71	32.51	1610.31	8.1
wool	192.32	2.67	7.63	4784.69	8.15
cotton	215.53	3.58	17.20	7272.73	7.45

**Table 2:** Physical properties grading criteria.

Physical Property	Grading Criteria (X is Normalized)				
Roughness	Level 1	Level 2	Level 3	Level 4	Level 5
Fullness	0 ≤ X ≤ 10	10 < X ≤ 20	20 < X ≤ 30	30 < X ≤ 40	40 < X ≤ 50
Bendability					
Elasticity	Level 6	Level 7	Level 8	Level 9	Level 10
Thermal-conductivity	50 < X ≤ 60	60 < X ≤ 70	70 < X ≤ 80	80 < X ≤ 90	90 < X ≤ 100

**Table 3:** Physical property fitting results.

Physical Property			Undetermined Parameters				R-Squared
$x_{1}$	$x_{2}$	$x_{3}$	$a_{1}$	$a_{2}$	$a_{3}$	b	
−*Kv*	Δ*K*	*K*	0.1357	0.1305	0.0788	60.5963	0.1693
Δ*K*	*K*	*Kw*	1.0978	0.0117	0.0143	68.4913	0.0386
*K*	*Kw*	*B*	0.1362	0.1048	0.4041	66.4129	0.2212
*Kw*	*B*	−*Kv*	0.2472	0.1816	0.2208	64.1700	0.4386
*B*	−*Kv*	Δ*K*	0.1717	0.1588	0.0647	63.4502	0.2411

## Data Availability

The data that support the findings of this study are available on request from the corresponding author.

## Supplementary Materials

The following supporting information can be downloaded at: https://www.mdpi.com/article/10.3390/s25103187/s1, Figure S1: Under the conditions of a light tent, participants explored the tactile sensations of a set of fabric samples by repeatedly rubbing their dominant index fingers. Table S1: The single exclusion method determines the degree to which physical properties affect the haptic feel of the fabric. In Equation (8), the undetermined parameters of the excluded physical properties are assigned to 0, and the smaller the R-squared, the greater the influence of the excluded items on the haptic feel of the fabric.
