# Supplementary material for: Fabric Tactile Prediction Method Based on Spider Diagram
Source: Sensors (Basel). 2025 May 19;25(10):3187. doi: 10.3390/s25103187 (PMC12115606; doi:10.3390/s25103187)

Figure S1. Under the conditions of a light tent, participants explored the tactile sensations of a set of fabric samples by repeatedly rubbing their dominant index fingers.

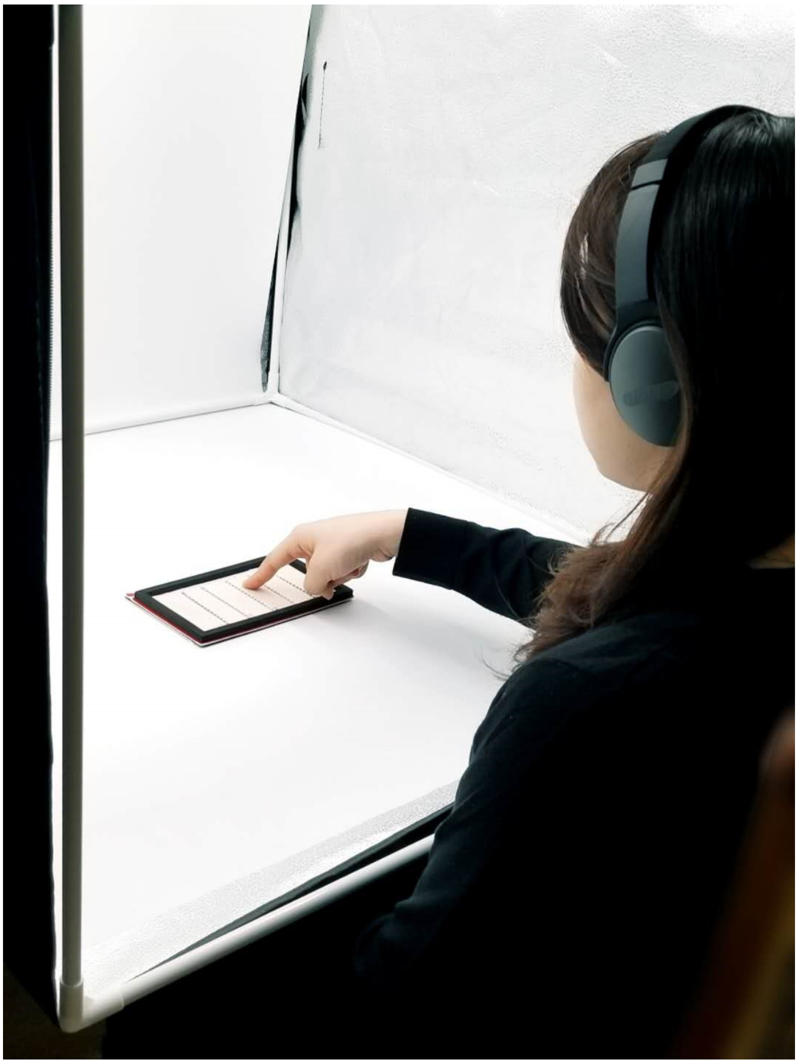

Supplement: Supplementary file 1 [file sensors-25-03187-s001.zip › Figure S1.pdf]
