# Supplementary material for: Fabric Tactile Prediction Method Based on Spider Diagram
Source: Sensors (Basel). 2025 May 19;25(10):3187. doi: 10.3390/s25103187 (PMC12115606; doi:10.3390/s25103187)
Supplement: Supplementary file 1 [file sensors-25-03187-s001.zip › Table S1.pdf]

Table S1. The single exclusion method determines the degree to which physical properties affect the haptic feel of the fabric. In equation (8), the undetermined parameters of the excluded physical properties are assigned to 0, and the smaller the R-squared, the greater the influence of the excluded items on the haptic feel of the fabric.

Exclusions	undetermined parameters						R-squared
	a_1	a_2	a_3	a_4	a_5	b	
-Kv	0	1.6452	0.0127	0.0393	1.1681	63.6696	0.2703
ΔK	0.2532	0	0.1298	0.2553	0.2208	55.0447	0.65379
K	0.1627	0.1400	0	0.3941	0.1486	54.2733	0.58499
Kw	0.0788	0.0650	0.3308	0	0.4859	59.1925	0.32994
B	2.8653	0.0078	0.0024	7.3417	0	57.4451	0.21291
